# Supercritical Carbon Dioxide-decellularized Porcine Acellular Dermal Matrix combined with Autologous Adipose-derived Stem Cells: Its Role in Accelerated Diabetic Wound Healing

**DOI:** 10.7150/ijms.41155

**Published:** 2020-02-04

**Authors:** Ping-Ruey Chou, Yun-Nan Lin, Sheng-Hua Wu, Sin-Daw Lin, Periasamy Srinivasan, Dar-Jen Hsieh, Shu-Hung Huang

**Affiliations:** 1 School of Medicine, College of Medicine, Kaohsiung Medical University, Kaohsiung 807, Taiwan; 2 Department of Anesthesiology, School of Medicine, College of Medicine, Kaohsiung Medical University, Kaohsiung 807, Taiwan; 3 Department of Anesthesiology, Kaohsiung Medical University Hospital, Kaohsiung 807, Taiwan; 4 Department of Anesthesiology, Kaohsiung Municipal Ta-Tung Hospital, Kaohsiung 807, Taiwan; 5 Division of Plastic Surgery, Department of Surgery, Kaohsiung Medical University Hospital, Kaohsiung 807, Taiwan; 6 Center of Research and Development, ACRO Biomedical Co., Ltd. Kaohsiung 821, Taiwan.; 7 Department of Surgery, School of Medicine, College of Medicine, Kaohsiung Medical University, Kaohsiung 807, Taiwan; 8 Regeneration Medicine and Cell Therapy Research Center, Kaohsiung Medical University, Kaohsiung 807, Taiwan

**Keywords:** acellular dermal matrix, supercritical carbon dioxide, autologous adipose-derived stem cells, diabetes mellitus, wound healing

## Abstract

Diabetes mellitus (DM) causes impaired wound healing by affecting one or more of the biological mechanisms of hemostasis, inflammation, proliferation, and remodeling and a large number of cell types, extracellular components, growth factors, and cytokines. Interventions targeted toward these mechanisms might accelerate the wound healing process. To evaluate the wound healing efficacy of supercritical carbon dioxide (scCO_2_)-decellularized porcine acellular dermal matrix (ADM) combined with autologous adipose-derived stem cells (ASCs) in streptozotocin (STZ)-induced DM rats. DM was induced by injecting rats with STZ; dorsal full-thickness skin (5 × 5 cm^2^) was created and treated with and without ASCs-scCO_2_-treated ADM to evaluate the wound healing rate through histological examination, fluorescence microscopic observation, and immunohistochemical analysis. In the present study, complete decellularization of the porcine dermal matrix was achieved through scCO_2_. Isolation of ASCs was conducted and evaluated using CD29^+^/CD31^-^/CD45^-^/CD90^+^ markers in flow cytometry, which indicated that more than 90% of cells were ASCs. The percentage of cells labeled with CD29^+^ and CD90^+^ was found to be 97.50% and 99.69%, respectively. The wound healing rate increased in all groups relative to the group with the DM wound without treatment. DM wound treated with ADM-ASCs showed significantly higher (*p* < 0.01) wound healing rate than DM wound without treatment. ADM-ASC-treated rats showed significantly increased epidermal growth factor, Ki67, and prolyl 4-hydroxylase and significantly decreased CD45 compared with the group with the DM wound without treatment. The intervention comprising ADM decellularized from porcine skin by using scCO_2_ and ASCs was proven to improve diabetic wound healing. ADM-ASCs had a positive effect on epidermal regeneration, anti-inflammation, collagen production and processing, and cell proliferation; thus, it accelerated wound healing.

## Introduction

Diabetes mellitus (DM) is a prevalent metabolic disease caused by impaired blood glucose control. DM affects approximately 350 million people worldwide, and the incidence of DM is still steadily increasing 1. Diabetic wounds are one of the complications of DM; delayed wound healing in the lower extremities is prevalent in 15%-25% of DM patients 2, 3. Diabetic wounds are chronic wounds 4; the normal wound healing process controlled by appropriate inflammation, cell proliferation and migration, and epidermal regeneration is impaired in DM patients 5. Diabetic wound healing is mainly delayed by prolonged inflammation 6, and the most severe consequence of delayed healing is amputation due to local infection and acral necrosis 7. The annual incidence of diabetic foot ulcers and amputation is estimated at 1%-4.1% and 0.21%-1.37%, respectively 8, 9. DM complications such as peripheral vascular disease, infection, and neuropathy necessitate multidisciplinary approaches to enhance diabetic wound healing 9, 10.

Adipose-derived stem cells (ASCs) are ideal pluripotent cells for producing various types of tissues through tissue engineering for clinical application 11. For instance, autologous ASCs and their clinical efficacy and safety in tissue regeneration have been investigated from breast reconstruction to neural regeneration in the spinal cord 12-14. Autologous ASCs accelerate wound healing in the treatment of chronic ulcers of lower extremities in peripheral artery disease, which is closely associated with DM 15. ASC injection around wound tissue was demonstrated to enhance healing through cell differentiation and angiogenesis 16. However, injected ASCs have exhibited poor distribution and a low survival rate for cell migration and proliferation during tissue regeneration 17, 18. To overcome this limitation, in tissue engineering, acellular dermal matrix (ADM) scaffolds were used as a carrier to enhance ASC distribution and survival to improve wound healing 19. ADM possess biocompatible and biodegradable properties; therefore, it can be used as a form of wound dressing for wound management 20. ADM is mainly composed of extracellular matrix (ECM), which comprises a wide range of biological factors that induce cell growth and regulate tissue responses, and the 3D microstructure of ECM in ADM serves as scaffolds for tissue engineering 21-23. The 3D structure offers tensile attachment positions for specific cells, cell surface receptors, or signaling factors that modulate the wound healing process 7, 24.

ADM is produced by decellularization through physical, chemical, or enzymatic methods to retain ECM from biological dermal tissues, such as human or cadaver skin, bovine dermis, and porcine skin 25. However, the existing decellularization methods used for ADM preparation are time consuming and involve chemicals that damage the components of ECM 26. Supercritical carbon dioxide (scCO_2_) extraction technology has proved to be an excellent alternative method because it is environmentally friendly, nontoxic, and efficient in eliminating toxic chemicals and retaining ECM components 22. Decellularization technology using scCO_2_ is advantageous, with no solvent residue, no off-odors, and complete solubilization and removal of hydrocarbons, including lipids. However, polar molecules such as proteins are preserved in scCO_2_ and can be removed by adding polar cosolvents such as ethanol. The scCO_2_ technique completely destroys and eliminates pathogens 27. Acellular porcine cornea (ABCcolla^®^ Collagen Ophthalmic Matrix, ACRO Biomedical Co., Ltd.) was produced employing scCO_2_ 28, which is used in this study and is now under evaluation in a human clinical trial in Taiwan.

Enhanced cell proliferation, growth, and differentiation were observed in scCO_2_-treated ADM with ASCs; thus, scCO_2_-treated ADM might be a key component of tissue engineering 22. Delivery of ASCs through ADM scaffolds accelerated diabetic wound healing through a paracrine mechanism, which enhanced granulation tissue formation and increased re-epithelialization and neovascularization 29. In this study, we seeded ASCs on scCO_2_-treated ADM from porcine skin. Our experimental manipulation aimed to ensure the efficient seeding and differentiation of ASCs and the intact native collagen structure of scCO_2_-treated ADM. Thus, we hypothesized that scCO_2_-treated ADM scaffolds might increase cell proliferation and regeneration, anti-inflammation, collagen recovery, and epidermal regeneration, thereby enhancing the wound healing efficacy in a diabetic rat model. In the present study, DM was induced by injecting rats with streptozotocin (STZ); dorsal full-thickness skin (5 × 5 cm^2^) was created and treated with and without ASC-scCO_2_-treated ADM for the evaluation of the wound healing rate through histological examination, fluorescence microscopic observation, and immunohistochemical (IHC) analysis.

## Materials and Methods

### Preparation of ADM scaffolds from porcine skin using scCO_2_

**Figure 1:**
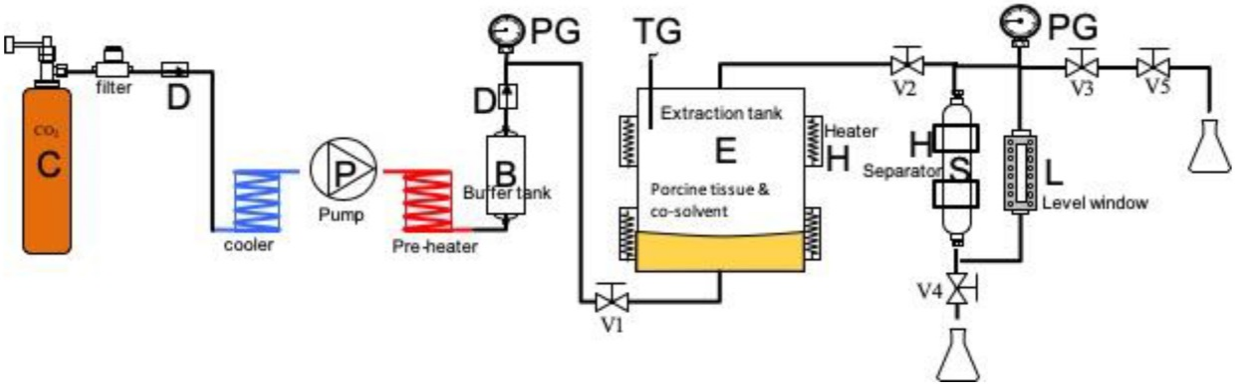
Schematic of the supercritical carbon dioxide extraction system for preparing ABCcolla^®^ Collagen Matrix: B, buffer tank; C, CO_2_ tank; D, check valve; E, extraction vessel; H, heater and jacket; L, level window; P, super-critical CO_2_ pump; S, separator; V1~4, high-pressure valves; V5, release valve; PG, pressure gauge; TG, temperature gauge. The porcine tissue and cosolvent were loaded into the extraction tank for each run. The extraction tank was heated using an electrical heater, and power was adjusted to provide temperatures of 30-50 °C. The system was pressurized to 20-35 MPa using carbon dioxide (Jing-De Gas, Kaohsiung, Taiwan). The process involved mixed-batch extraction and continuous extraction. After completion of extraction, the high-pressure valves V3, V4, and V5 were opened to release pressure and separate the waste.

**Figure 2:**
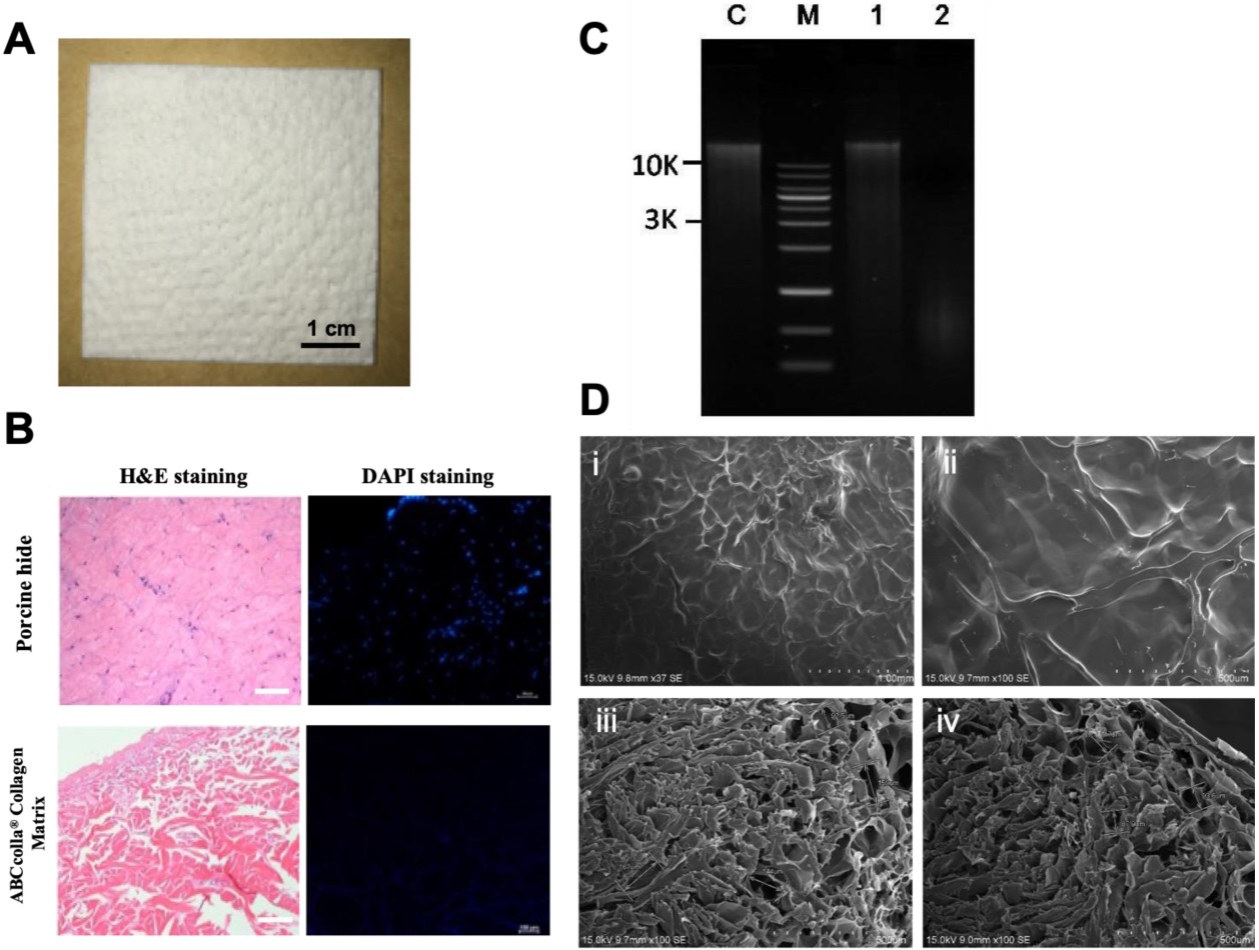
Representative macroscopic and microscopic images of ABCcolla^®^ Collagen Matrix. **(A)** Macroscopic view of ABCcolla^®^ Collagen Matrix. Scale bar = 1 cm. **(B)** Hematoxylin and eosin staining and 4,6-diamidino-2-phenylindole (DAPI) fluorescent staining of normal skin and ABCcolla^®^ Collagen Matrix, which show no cell in ABCcolla^®^ Collagen Matrix. Scale bar = 100 μm. **(C)** Agarose gel electrophoresis for quantifying DNA. Lane M: GMM002 1-kb DNA ladder marker, Lane C: GM-control fish tissue, Lane 1: No. 1 Porcine Skin, Lane 2: No. 2 Acellular Dermal Matrix; 0.8% agarose gel, 0.5X TAE (~300 ng sample loading). **(D)** SEM photomicrographs: (i & ii) Superficial view, and (iii & iv) cross-section of ABCcolla^®^ Collagen Matrix.

Porcine skin was purchased from Tissue Source, LLC (Lafayette, Indiana 47909, USA). Residual fat tissues were cleaned from the skin, and the skin was washed with phosphate-buffered saline (PBS). ADM (ABCcolla^®^ Collagen Matrix, ACRO Biomedical Co. Ltd, Kaohsiung, Taiwan) (Fig. 2A) was prepared from the porcine dermal layer. Dermal layers were physically detached from the porcine skin, after which scCO_2_ extraction was performed for complete decellularization. The flow chart is presented (Fig. 1). The skin was thinly sliced to 0.7-1.0-mm-thick sections; the sections were placed in a tissue holder, which was then placed into a scCO_2_ vessel system (Helix SFE Version R3U, Applied Separations Inc., Allentown, PA, USA); in the vessel, 10 mL of 75% ethanol was added as a cosolvent. The scCO_2_ system was then operated at 200-350 bar and 30°C-50°C for 40 min to produce ADM. ADM was washed in (1%-10%), followed by washing in hydrogen peroxide (H_2_O_2_, 10%-35%) and finally washing in sodium hydroxide (0.1-1 N), and ADM was freeze-dried and sterilized by γ-irradiation (25 kGy); the product used for this study is marketed as ABCcolla^®^ Collagen Matrix.

### Verification of the complete decellularization of ADM scaffolds

ADM scaffolds and native porcine skin were fixed in 4% buffered formaldehyde, and the sections stained with hematoxylin and eosin (H&E) to evaluate complete decellularization. The stained sections were imaged under a microscope (Olympus BX53).

Paraffin-embedded sections were cut to 5-µm thickness, dewaxed in xylene, and rehydrated in a graded series of alcohol to water. The sections were stained with 4,6-diamidino-2-phenylindole (DAPI), and images were obtained under a fluorescent microscope. DNA was analyzed semiquantitatively through standard agarose gel electrophoresis to confirm the removal of cells in decellularized ADM. Scanning electron microscopy was performed to verify the porous nature of decellularized ADM.

### Isolation, surface marker analysis, labeling, and transplantation of ASCs

Autologous adipose tissues were harvested from the left inguinal areas of rats and were minced (approximately 1 × 1 × 2 cm^3^ in size) using scissors. Tissues were washed in PBS and digested with 0.075% collagenase (37.5 mg/mL; Sigma-Aldrich, St. Louis, MO, USA) in PBS at 37°C with constant agitation for 30 min. The digested samples were then centrifuged (at 800 ×g for 10 min) to separate the supernatant containing mature adipocytes from the pellet. The pellet was suspended and filtered through a 40-mm cell strainer (BD Biosciences, Franklin Lakes, NJ, USA), washed and incubated in Dulbecco's modified Eagle's medium (GIBCO/Invitrogen Corporation, Carlsbad, CA, USA), and supplemented with 2 mmol/L N-acetyl-L-cysteine (Sigma-Aldrich) and 0.2 mmol/L L-ascorbic acid 2-phosphate (Sigma-Aldrich) at 37°C. The medium was changed every 3 days.

After culturing for a week, cells were trypsinized, washed twice with PBS, blocked for 1 h at room temperature, and incubated overnight at 40°C with a solution of fluorescein isothiocyanate (FITC)-conjugated or R-phycoerythrin (PE)-conjugated antibodies in PBS. A flow cytometry system (LSR II, BD Biosciences) was used to examine the expression of surface markers on subcultured cells collected during the third passage. The following antibodies were used: FITC anti-mouse/rat CD29 (1:200 dilution, 102206, BioLegend, San Diego, CA, USA), FITC anti-rat antibodies CD90 (1:200 dilution, 206106, BioLegend), FITC mouse anti-rat CD31 (1:200 dilution, MCA1334, Serotec, Raleigh, NC, USA), and PE anti-rat CD45 (1:200 dilution, 202207, BioLegend). The first two of these antibodies were used as positive antigen markers and the second two as negative antigen markers. FITC rat immunoglobulin (1:200 dilution, IgG2b), k isotype control and PE rat IgG2a, and k isotype control (1:200 dilution, 400508, BioLegend) were used as staining controls and to ensure accurate measurements. ASCs were defined as cells that were CD29^+^/CD31^-^/CD45^-^/CD90^+^ on flow cytometric analysis.

ASCs were labeled with chloromethylbenzamido (C7000, CellTracker CM-DiI; Invitrogen Life Technologies, Carlsbad, CA, USA) according to the manufacturer's instructions. Labeled cells from the fourth passage were visualized using fluorescence microscopy prior to harvesting for treatment. The engraftment of cultured ASCs into the full-thickness wound was identified by the colocalization of chloromethylbenzamido dialkylcarbocyanine (CM-DiI). ASCs were labeled with CM-DiI (C7000, CellTracker CM-DiI; red) and DAPI nuclear counterstaining (blue) and were evaluated through fluorescence microscopy.

### Animal wound healing experiment

#### Animal group and STZ-induced diabetic rats

Male Wistar rats (n = 24) weighing 300-350 g were used in this study. Rats were injected with freshly prepared STZ (Sigma-Aldrich) 65 mg/mL solution in 0.1 M citrate buffer (pH = 4.5) at a dose of 60 mg/kg i.p. Roche Accu-Chek was applied to measure blood glucose after 72 h using the blood collected from the tail vein of rats for verifying the induction of DM (glucose >300 mg/dL). The study protocol was approved by the Institutional Animal Care and Use Committee of Kaohsiung Medical University (IACUC Approval Number: 105015). All rats were housed in plastic cages with soft bedding under 12-h light/dark cycles, with free access to food and water. Rats were randomly divided into four groups (n = 6 per group) as follows: the diabetic control group (DM wound, DM-), in which the dorsal skin defect in diabetic rats was created without any treatment; the group of diabetic rats treated with ADM dressing (ABCcolla^®^ Collagen Matrix) (DM + ADM); the group of diabetic rats treated with ASCs at 1 × 10^8^ cells in a 1-mL injection per area (DM + ASCs); and the group of diabetic rats treated with ADM dressing seeded with ASCs at 1 × 10^8^ cells (DM + A/A) (Fig. 4B). The wound was then temporarily covered with transparent DuoDerm Extra Thin CGF Dressing (ConvaTec Inc.).

#### Wound healing animal model

DM was induced using STZ; 1 week later, Zoteil 50 i.p. was used for anesthesia. The dorsum of rats was shaved, and a 5 × 5 cm^2^ patch was drawn on the dorsal skin using a marker pen. The palpable hip joints were used as anatomical landmarks for defining the base of the wound defect. Incisions were made under sterile conditions, and the entire flap was undermined below the level of the dorsal fascia. The skin flap was excised to create a skin defect with an area of 5 × 5 cm^2^. The margin of the wound defect was sutured in place with 4-0 silk sutures to prevent wound contracture after being covered by ABCcolla^®^ Collagen Matrix dressing and transparent DuoDerm (ConvaTec Inc.).

#### Wound healing area estimation

The wound healing area was assessed every 7 days (1 week) after operation by using the described template technique. The healed area was calculated as [1 - (A1/A0) × 100%] from the original wound area (5 × 5 cm^2^ as A0) and the unhealed area (A1) every 7 days until the wound had completely healed.

### Histological examination

The full-thickness wound margin was harvested on day 42 (end of the sixth week) from the animals and was fixed in 10% formaldehyde. The samples were embedded in paraffin and sectioned perpendicular to the epidermal surface into 4-μm-thick slides. Each section was deparaffinized in xylene and stained with H&E; Masson trichrome staining was conducted according to the routine histologic protocol. The outcomes of collagen recovery and epidermal reconstruction were observed through H&E and Masson trichrome staining. The percentage of collagen deposition and the number of cells per high-power view in the tissue were quantified using Image-Pro Plus Version 6.0 software (Media Cybernetics, Inc., Rockville, MD, USA).

### IHC assay

Paraffin-embedded 4-μm-thick tissue sections were deparaffinized with xylene and rehydrated with a gradient of decreasing ethanol (100%, 95%, 70%, and 50%) at room temperature. Endogenous peroxidase (PO) was blocked by placing the sections in H_2_O_2_ for 5 min. The sections were then incubated with antiepidermal growth factor (EGF; 1:400 dilution; Novus Biologicals, LLC, Centennial, CO, USA), antivascular EGF (VEGF; 1:400 dilution; Novus Biologicals), anti-CD45 (1:400 dilution; Abcam, Cambridge, UK), anti-Ki67 (1:200 dilution; Novus Biologicals), and anti-prolyl 4-hydroxylase (P4HA1; 1:400 dilution; Mybiosource, San Diego, CA, USA) sequentially. The sections were incubated with the primary antibody at room temperature for 60 min. The sections were then washed with tris-buffered saline and incubated with the biotinylated secondary antibody Histofine^®^ Simple Stain Rat MAX PO (Nichirei Bioscience, Nichirei Corporation, Tokyo, Japan) for 1 h. Histofine^®^ Simple Stain Rat MAX PO is a labeled polymer prepared by combining amino acid polymers with PO and rabbit anti-goat IgG, which are reduced to the Fab fragment. The diaminobenzidine substrate was added and allowed to react for 5 min (Dako Products, Santa Clara, CA, USA). Finally, the dye hematoxylin was added. The section was quantified using Image-Pro Plus Version 6.0 software (Media Cybernetics, Inc.).

### Statistical analysis

The experimental results are presented as mean ± standard deviation (SD). One-way analysis of variance was performed to determine the significant differences in groups with a normal distribution. Post hoc comparison was performed using Tukey's HSD tests, and * p < 0.05, ** *p* < 0.01, and *** *p* < 0.001 were considered statistically significant.

## Results

### Decellularization and structure of ADM scaffolds

To verify the complete decellularization of the dermal matrix, H&E staining was conducted. H&E staining depicted no cellular components in decellularized ADM. In native porcine skin, the nucleus was stained by hematoxylin, appearing as blue-purple color, and eosin bound to the protein in the cytoplasm, appearing as pink color. Therefore, H&E staining indicated the complete decellularization of ADM (Fig. 2B). DAPI binds to the nucleus or DNA and emits a blue color under fluorescent light. However, in the present study, scCO_2_-decellularized ADM scaffolds showed no obvious nucleus, confirming the complete decellularization of ADM scaffolds (Fig. 2B; scale bar = 100 μm). To verify complete decellularization, residual DNA was analyzed (Fig. 2C; lane C, control fish tissue; lane M, marker of DNA; lane 1, porcine skin showing the normal amount of DNA; and lane 2, ADM, showing very faint band). The figure indicates the presence of a tiny amount of DNA (below 50 ng/mg) in ADM. SEM photographs of decellularized ADM revealed fibrous interconnected networks of collagen fibers in the superficial view (Fig. 2D, i and ii); these fibrous interconnected networks of collagen fibers might attach to the wound site, forming a bottom layer, and cells were added on top of decellularized ADM. The cross-section of decellularized ADM depicted pores that were tunnels of interconnected tubes in varied pore sizes ranging from 80 to 160 µm. However, smaller pores constituted the whole cross-section of decellularized ADM (Fig. 2D, iii and iv).

### ASC flow cytometric analysis and engraftment

**Figure 3:**
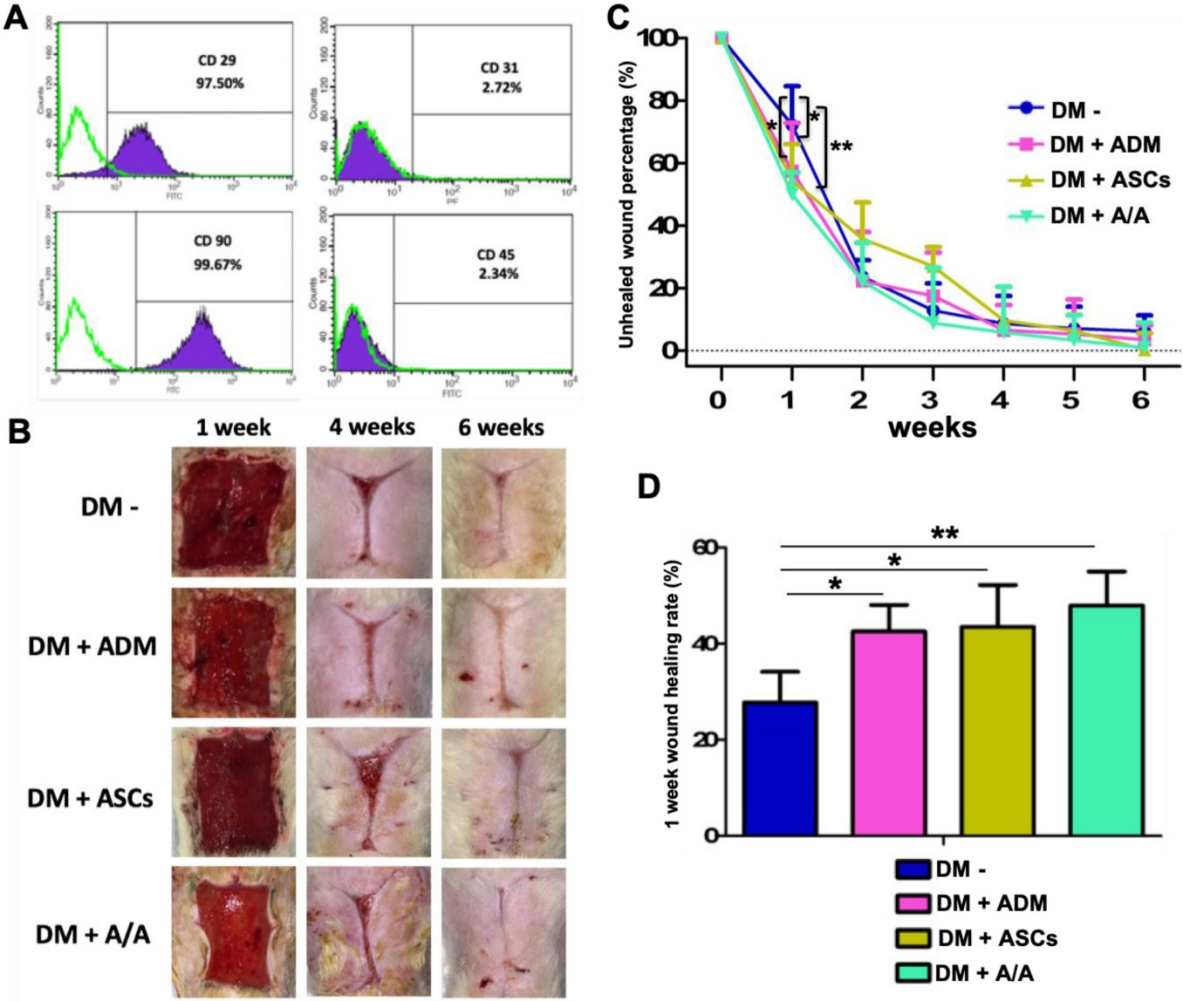
Wound healing accelerated by ASCs seeding into ABCcolla^®^ Collagen Matrix in the first week. **(A)** Surface marker analysis of ASCs. Flow cytometry results of rat ASCs. CD29+/CD31^-^/ CD90+/ CD45^-^ expression indicated the presence of ASCs. **(B)** Macroscopic wound healing photographs. **(C)** The ASC-ABCcolla^®^ Collagen Matrix (DM+A/A) group showed significantly decreased unhealed wound percentage compared with other groups in the first week (* p < 0.05, ** *p* < 0.01). **(D)** In the DM wound without treatment (DM-), the wound healing rate was significantly lower than that in the other groups in the first week (* p < 0.05, ** *p* < 0.01).

To determine the percentage of isolated ASCs, flow cytometric analysis was conducted using the CD surface marker. Cells were labeled with CD29^+^/CD31^-^/CD45^-^/CD90^+^ antibodies. The percentage of cells labeled with CD29^+^ and CD90^+^ was found to be 97.50% and 99.69%, respectively. The percentage of cells labeled with CD31^-^ and CD45^-^ was found to be 2.72% and 2.34%, respectively (Fig. 3A).

**Figure 4:**
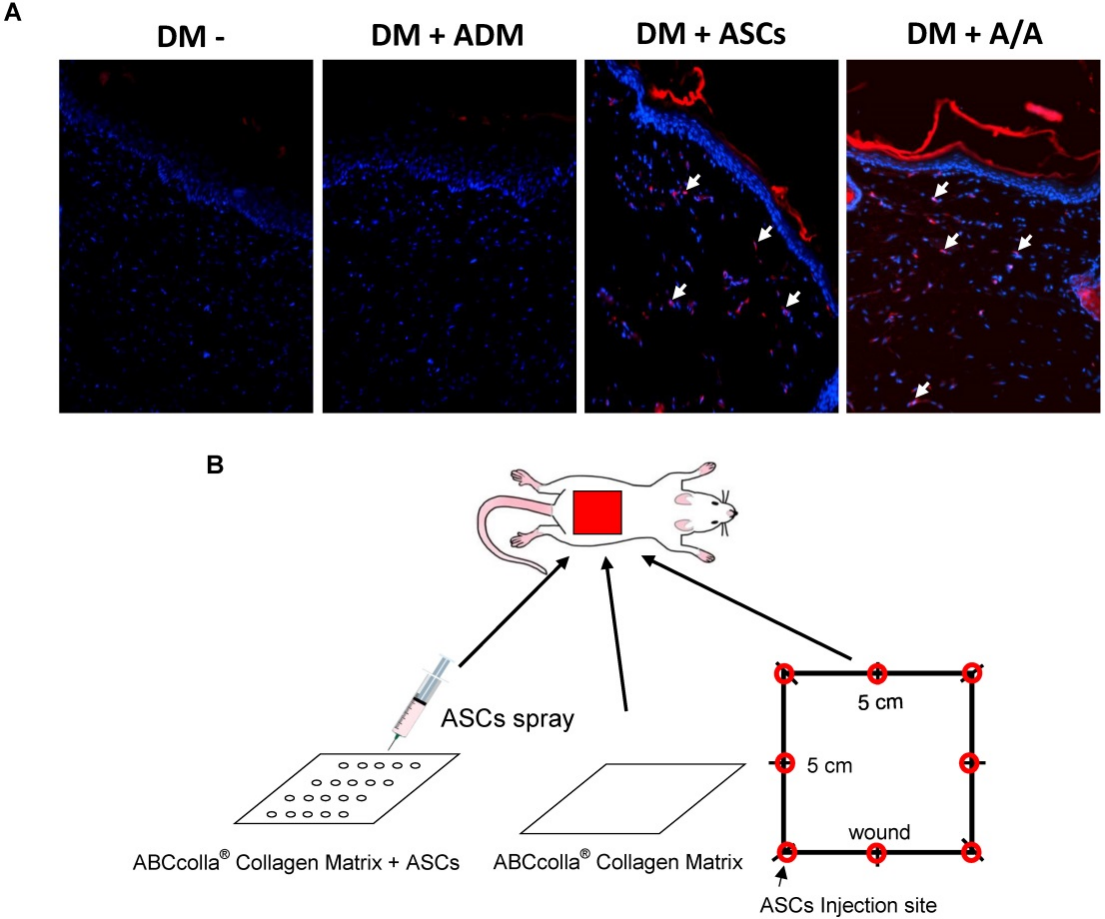
Engraftment of ASCs into the rat back dermal layer. **(A)** ASCs are indicated as red in CM-DiI and DAPI-positive nuclear fluorescent staining, and the arrow indicates double-positive cells in the rat back dermal layer. **(B)** Schematic of ASCs seeded into ABCcolla^®^ Collagen Matrix.

To track the movement of ASCs, the fluorescent dye CM-DiI was used for monitoring the cell location (Fig. 4A). CM-DiI has been shown to be effective for multigenerational tracking of cellular location. In the present study (Fig. 4A), rats with the DM wound (DM-) and with the DM wound treated with ADM alone (DM + ADM) did not show any CM-DiI fluorescence (red color), indicating no ASCs in the wound healing location. However, rats with the DM wound treated with ASCs alone (DM + ASCs) and with the DM wound treated with ADM-ASCs (DM + A/A) showed significant CM-DiI fluorescence, indicating the presence of different generations of ASCs that migrated inside the skin layer and were involved in the accelerated healing of the diabetic wound.

### Promotion of diabetic wound healing by ADM scaffolds plus ASCs

At the end of the sixth week, microscopic photomicrographs of the DM wound treated with ADM-ASCs (DM + A/A) showed more favorable wound healing outcomes than other experimental treatments (Fig. 3B). The healing area was measured and calculated every 7 days (Fig. 3C). At the end of the first week, the wound healing rate of all the experimental groups was significantly higher (*p* < 0.05) than that of the group with the diabetic wound without treatment (DM-) (Fig. 3D). The wound healing rate of the group with the DM wound treated with ADM-ASCs (DM + A/A) was significantly higher (*p* < 0.01) than that of the group with the DM wound without treatment. No significant difference was observed in the wound healing rate between the groups with the DM wound treated with ADM alone (DM + ADM), ASCs alone (DM + ASCs), and ADM-ASCs (DM + A/A).

### Upregulation of collagen recovery, epidermal regeneration, anti-inflammation, and cell proliferation and regeneration by ADM scaffolds plus ASCs

**Figure 5:**
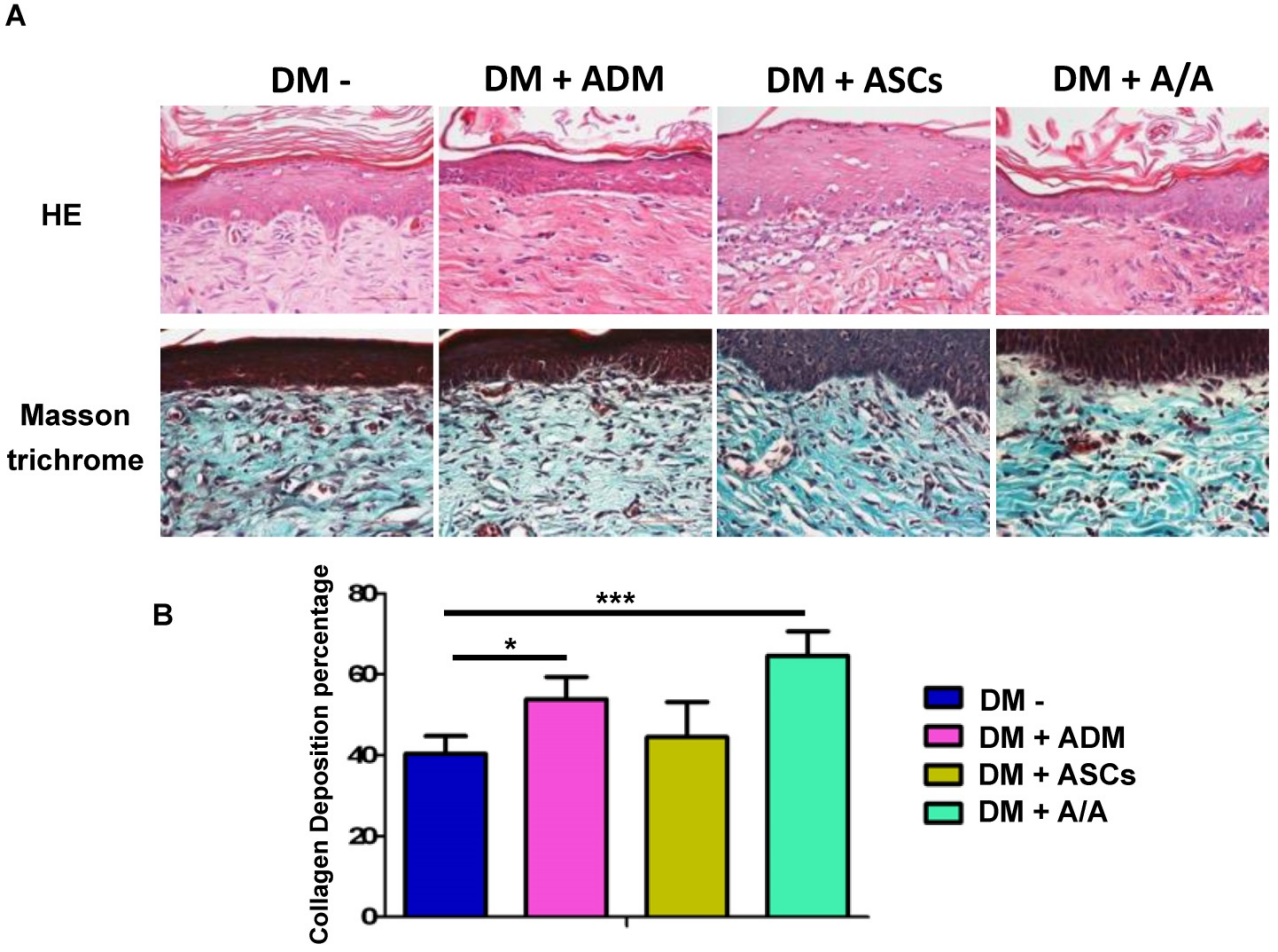
Collagen deposition significantly increased by ASCs-ADM. **(A)** Representative image of H&E and Masson trichrome staining, and **(B)** collagen deposition percentage of the group with the DM wound treated with ADM alone (DM + ADM) was significantly higher (*p* < 0.05) than that of the group with the diabetic wound without treatment (DM-). The group with the DM wound treated with ADM-ASCs (DM + A/A) showed a significantly higher (*p* < 0.001) collagen deposition percentage compared with the group with the DM wound without treatment (DM-). Scale bar = 50 μm.

**Figure 6:**
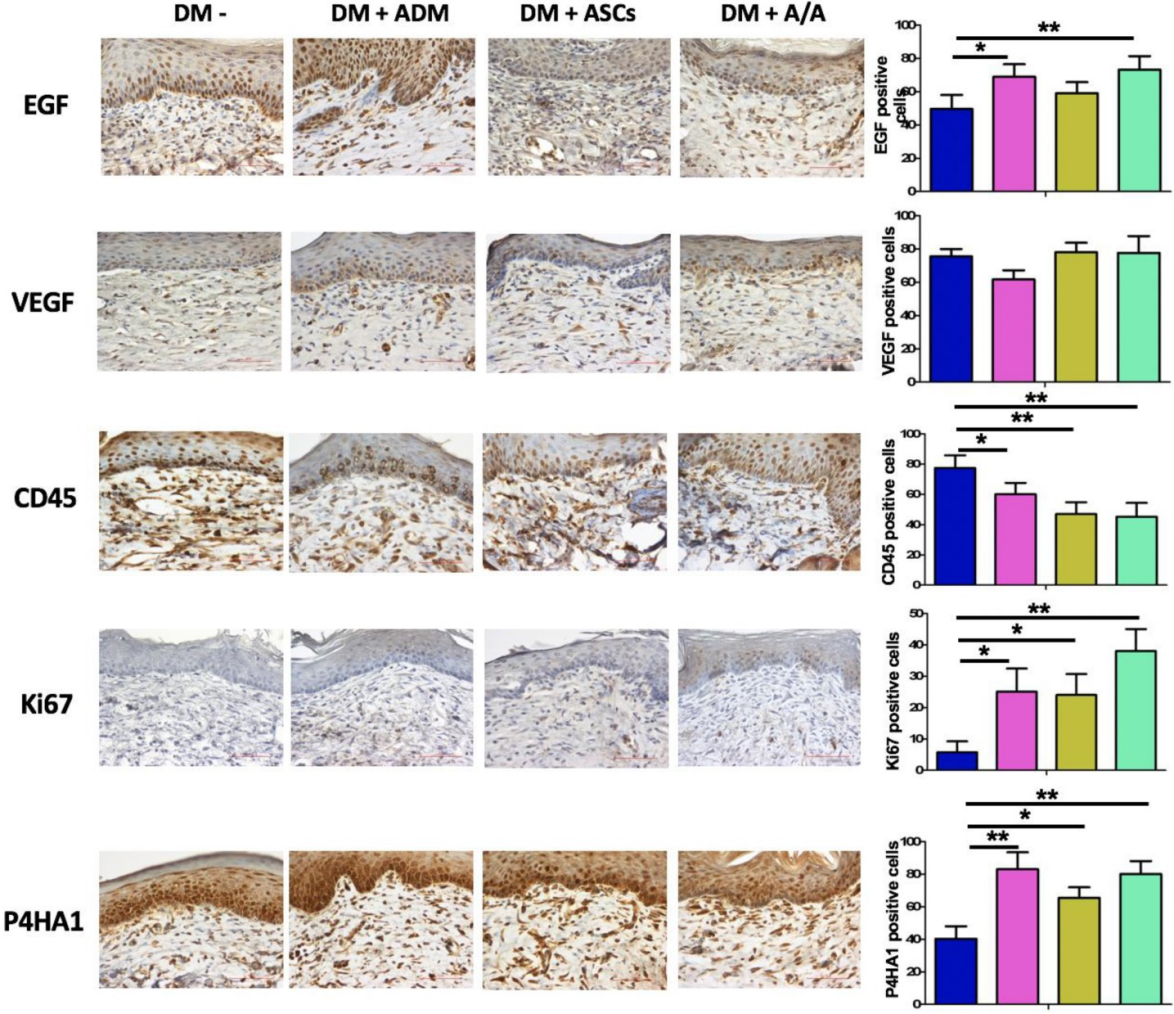
Representative images of EGF, VEGF, CD45, Ki67, and P4HA1 immunohistochemical expression and comparison. Scale bar = 50 μm.

Collagen recovery in the wound healing process is highly related to collagen deposition and production. In the present study, histopathology (Fig. 5A) revealed the group with the DM wound treated with ADM-ASCs (DM + A/A) showed obvious collagen deposition compared with the groups with the DM wound without treatment (DM-), DM wound treated with ASCs alone (DM + ASCs), and DM wound treated with ADM-ASCs (DM + A/A) (scale bar = 50 μm). The collagen deposition percentage (Fig. 5B) of the group with the DM wound treated with ADM alone (DM + ADM) was significantly higher (*p* < 0.05) than that of the group with the diabetic wound without treatment (DM-) (scale bar = 50 μm). The collagen deposition percentage of the group with the DM wound treated with ADM-ASCs (DM + A/A) was significantly higher (*p* < 0.001) than that of the group with the DM wound without treatment (DM-). No significant difference was observed in the collagen deposition percentage between the groups with the DM wound treated with ASCs alone (DM + ASCs) and the DM wound without treatment (DM-). P4HA1 is an enzyme highly associated with collagen biosynthesis. In this study, the expression of P4HA1 in all the experimental groups was significantly higher (*p* < 0.05, *p* < 0.01) than that in the group with the diabetic wound without treatment (DM-) (Fig. 6). P4HA1 expression was significantly increased (*p* < 0.01) in the group with the DM wound treated with ADM-ASCs (DM + A/A) compared with the group with the DM wound without treatment (DM-) (Fig. 6, Table 1). No significant difference was observed in the elevated expression of P4HA1 between the groups with the DM wound treated with ADM alone (DM+ADM), DM wound treated with ASCs alone (DM+ASCs), and DM wound treated with ADM-ASCs (DM + A/A).

Epidermal regeneration can be enhanced by EGF-stimulated keratinocyte proliferation and migration. In this study, EGF was found to have immunomodulatory effects that reduced the inflammatory response through the downregulation of chemokines and cytokines secreted by keratinocytes. The expression of EGF was significantly increased (*p*<0.05, *p* < 0.01) in the groups with the DM wound treated with ADM alone (DM + ADM) and DM wound treated with ADM-ASCs (DM + A/A) compared with the group with the diabetic wound without treatment (DM-) (Fig. 6). EGF expression was significantly increased (*p* < 0.01) in the group with the DM wound treated with ADM-ASCs (DM + A/A) compared with the group with the diabetic wound without treatment (DM-). Furthermore, EGF expression was highest in all experimental groups (scale bar = 50 μm; Fig. 6, Table 1). No significant difference was observed in EGF expression between the group with the diabetic wound without treatment (DM-) and the group with the DM wound treated with ASCs alone (DM + ASCs). CD45 is associated with inflammatory response along with fibroblast activation. The expression of CD45 in all the experimental groups was significantly lower (*p* < 0.05, *p* < 0.01) than that in the group with the diabetic wound without treatment (DM-) (Fig. 6). The group with the DM wound treated with ADM-ASCs (DM + A/A) showed significant decreases (*p* < 0.01) in CD45 expression compared with the group with the diabetic wound without treatment (DM-), and CD45 expression was the lowest in all experimental groups (Fig. 6, Table 1). No significant alteration in the expression of CD45 was detected between the groups with the DM wound treated with ADM alone (DM + ADM), DM wound treated with ASCs alone (DM + ASCs), and DM wound treated with ADM-ASCs (DM + A/A).

Ki67 is a cell proliferation and regeneration marker. In this study, the expression of Ki67 in all the experimental groups was significantly higher (*p* < 0.05,* p* < 0.01) than that in the group with the diabetic wound without treatment (DM-) (Fig. 6). The group with the DM wound treated with ADM-ASCs (DM + A/A) showed significant increases (*p* < 0.01) in Ki67 expression compared with the group with the DM wound without treatment (DM-), and Ki67 expression was the highest in all experimental groups (Table 1). No significant difference was observed in the expression of Ki67 between the groups with the DM wound treated with ADM alone (DM + ADM), DM wound treated with ASCs alone (DM + ASCs), and DM wound treated with ADM-ASCs (DM + A/A).

### Evaluation of angiogenesis effect based on VEGF expression

The angiogenesis effect during wound healing can be evaluated based on VEGF expression. In the present study, higher VEGF expression was found in the group with the DM wound treated with ADM-ASCs (DM + A/A) than in the group with the DM wound treated with only ADM (DM + ADM) (Fig. 6, Table 1). However, no significant alteration in VEGF expression was found in all experimental groups.

## Discussion

The wound healing process involves several cellular and extracellular interactions that occur through a well-orchestrated cascade that includes inflammation, re-epithelialization, angiogenesis, granulation tissue formation, wound contraction, and tissue maturation 30, 31. In chronic wounds, abnormal and dysregulated interactions occur between growth factors, ECM, and other cellular constituents, which are all crucial in modulating the classic wound healing process 5. Diabetic wounds are chronic and heal poorly; the most severe consequence of delayed healing is amputation. Furthermore, the recurrence rate of diabetic wounds has been reported to be 60%, which is the highest rate among various types of chronic wounds 32. Inadequate blood supply results from peripheral vascular disease, which is a macrovascular complication of DM; this inadequate blood supply causes additional cellular and systemic stress to the microenvironment, leading to difficulty in wound healing 6, 33. Therefore, advanced diabetic wound care management based on debridement, improvement of vascular perfusion, infection control, and tissue engineering has been in development for years 34. In the present investigation, scCO_2_-processed ADM from porcine skin was tissue-engineered with ASCs, and the ADM-ASCs treatment was applied for diabetic wounds, which generally exhibit poor healing. Thus, scCO_2_-processed ADM from porcine skin provides native collagen scaffolds and thus can play a significant role in the acceleration of diabetic wound healing.

ASCs are a type of mesenchymal stem cells (MSCs), and they play a pivotal role in the field of tissue engineering 35. Adipose tissue is a major source of MSCs; subcutaneous adipose tissue contains abundant MSCs, with easy access and low morbidity 11, 36. ASCs can be extracted from the autologous stromal vascular fraction using enzymatic methods, and specific surface markers such as CD29^+^/CD31^-^/CD45^-^/CD90^+^ can be detected through flow cytometry to confirm that the isolated cells are ASCs 11. The survival of ASCs can be confirmed by CM-DiI labeling in immunofluorescent analysis 37. ASCs can be used for soft tissue regeneration through differentiation into specialized cells 11. Using the ASC-based approach, vascular, neural, muscular, and cartilage tissue defects can be repaired, and tissue functions can be restored 38-41. ASCs improve wound healing efficacy by producing signaling molecules and enhancing differentiation into required cells involved in wound healing 42. Cytokines such as transforming growth factor-beta, VEGF, hepatocyte growth factor, and granulocyte/macrophage colony-stimulating factor secreted by ASCs exert paracrine effects, facilitating wound healing 43, 44. In addition, the injection of autologous ASCs for wound management resulted in a decreased complication rate and a higher survival rate for full-thickness skin grafts 45. In diabetic wounds, injecting autologous ASCs into the edges of the wound resulted in excellent healing 46. In the present study, to confirm that the isolated cells were ASCs, cell markers such as CD29^+^/CD31^-^/CD45^-^/CD90^+^ were detected; the survival of ASCs injected into the diabetic wound was confirmed by CM-DiI labeling. ASC-injected DM rats (DM + ASCs) showed more favorable wound healing outcomes and higher Ki67 and P4HA1 expression than nontreated DM rats (DM-).

ADM is a biomaterial decellularized from animal or human skin/tissues 6. Structural, mechanical, and biochemical functions of ADM are attributed to ECM, which is the main component of ADM 47. Collagen I is the major element of ECM, and it is responsible for modulating cellular functions in the wound healing process 48. In chronic wounds, type I collagen can prevent the deterioration of wounds by binding to free radicals, proteases, and inflammatory cytokines in the wound bed 49. Therefore, the collagen-rich ECM in ADM plays a significant role in chronic wound healing. Several collagen-based scaffolds are produced from decellularized animal or human skin/tissues for DM patients, such as Integra^™^ (bovine collagen-derived), Oasis^®^ (swine small intestine submucosa-derived), and Epifix^®^ (human placenta-derived) 6. ADM from porcine skin is an ECM-rich scaffold widely used in tissue engineering 50. ADM can be used as a dressing to cover the wound and prevent infections. In addition, it is bioabsorbable and promotes wound healing due to its biodegradable and biocompatible properties 25. In the present study, we used ABCcolla^®^ Collagen Matrix, a wound dressing based on porcine skin-derived ADM, and it was found to accelerate diabetic wound healing in DM rats.

Decellularization technology can be employed to eliminate the antigenicity of xenogeneic tissues and retain the components of ECM. Decellularization techniques based on physical, chemical, or enzymatic approaches are performed by adding detergents, such as Triton-X100, sodium dodecyl sulfate (SDS), and sodium deoxycholate 51. However, detergents induce cytotoxicity. In particular, SDS was found to cause irreversible structural damage to ECM, and the decellularization technique based on SDS was time consuming 52. Decellularization technology using scCO_2_ is an alternative cost-effective method, is considered clean and green technology 22, and can be used to eliminate fat and cellular components and retain intact ECM without damage 50. The decellularized ECM structure and its components are well preserved, as confirmed by histological and IHC staining techniques 22. CO_2_ is commonly used as a supercritical fluid due to its appropriate critical temperature for processing ECM. In addition, it is an ambient gas with easy availability and application 50, 53. Furthermore, scCO_2_ decellularization technology is nontoxic, cheap, and environmentally friendly 11, 22. Fat reduction and removal from porcine skin can be conducted efficiently using the scCO_2_ method 50. In the present study, we prepared ABCcolla^®^ Collagen Matrix, a porcine skin-based ADM, by using scCO_2_ technology; its structural nativity was confirmed by H&E staining. Compared with the group with the DM wound without treatment (DM-), the group with the DM wound treated with ADM (DM +ADM) showed more favorable clinical outcomes of wound healing, as revealed by macroscopic observation and Ki67 expression, and more favorable collagen recovery of wounds, as determined through H&E staining, Masson's trichrome staining, and P4HA1 expression. In addition, the acceleration of epidermal regeneration and anti-inflammation induced by ABCcolla^®^ Collagen Matrix were evaluated through EGF and CD45 IHC analysis.

Conventional *in vivo* injection of stem cells results in incomplete ASC utilization due to poor biodistribution and low cell survival during wound healing 29. To overcome these limitations, tissue engineering studies have suggested the use of ADM as a scaffold for the delivery of ASCs into the wound healing microenvironment 21. The 3D structure of ECM in ADM scaffolds provides tensile attachment niches for specific cells, cell surface receptors, or signaling factors that modulate the wound healing process 7, 24. ADM scaffolds were found to be biocompatible, as assessed by NIH3T3 cells stained with DAPI 54. Autologous ASCs seeded onto the ADM-based dressing were found to be efficient for soft tissue regeneration 55. Targeted delivery of ASCs using ADM promoted growth and proliferation in the diabetic wound 29. In the present study, ASCs were seeded on ADM, to assess diabetic wound healing in a DM rat model. According to macroscopic observation, ADM-ASCs-treated rats (DM + A/A) exhibited enhanced wound healing. In addition, the CM-DiI cell tracker dye indicated that the group with the DM wound treated with ADM-ASCs (DM + A/A) exhibited CM-DiI fluorescence, indicating the presence of different generations of ASCs that migrated inside the skin layer and were involved in the accelerated healing of the diabetic wound and epidermal regeneration, cell proliferation with tissue remodeling, and collagen deposition.

**Figure 7:**
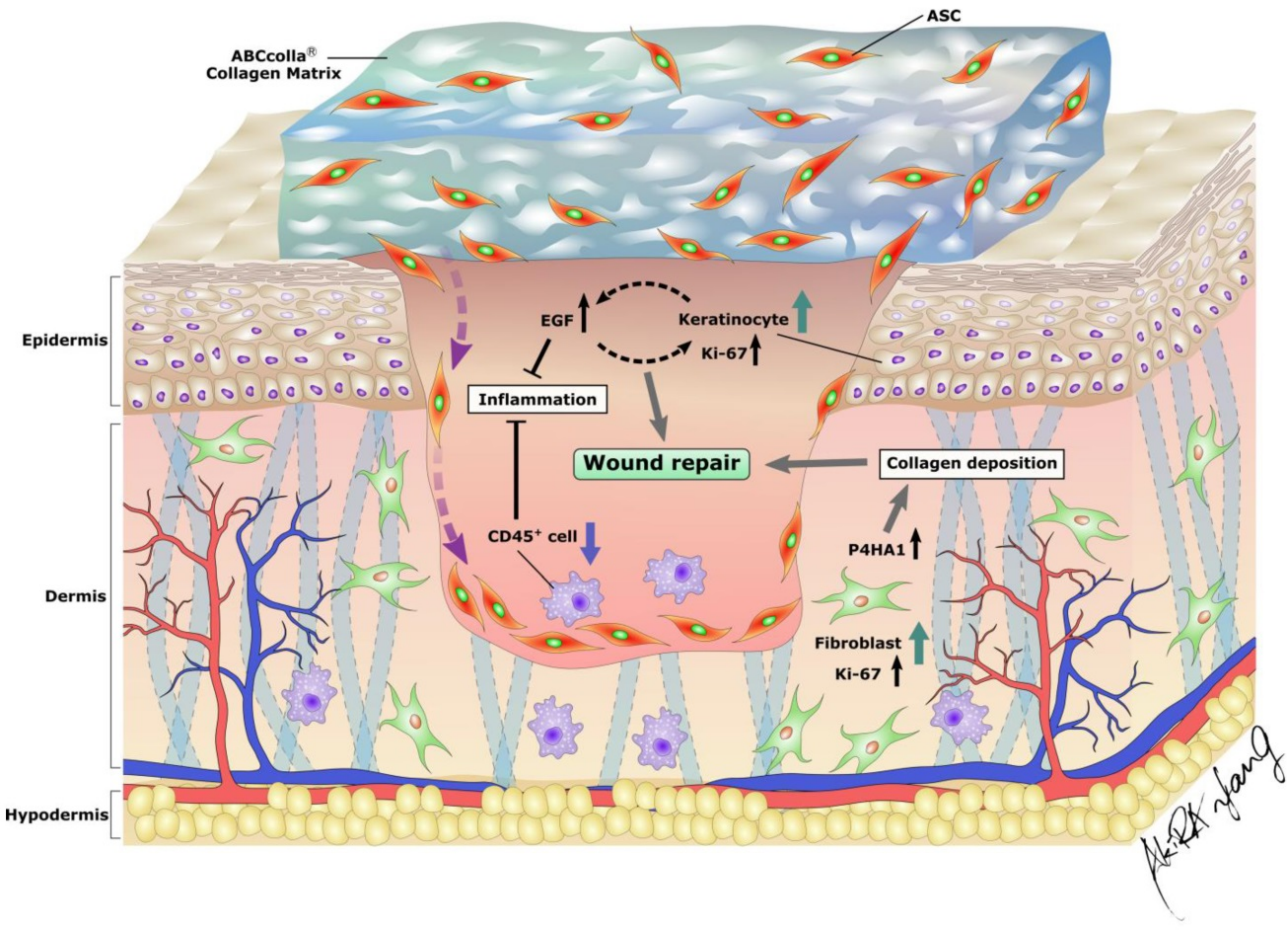
Excellent wound coverage and an optimal microenvironment for ASCs in diabetic wounds provided by ABCcolla^®^ Collagen Matrix. Combination of the collagen scaffold and ASCs accelerated the wound repair process by increasing the proliferation of both keratinocytes and fibroblasts and modulating epidermal regeneration, anti-inflammation, collagen production, processing, and cell proliferation in diabetic wounds in rats.

EGF secreted by keratinocytes plays a crucial role in epithelial homeostasis and wound healing. In addition, EGF has an immunomodulatory role that reduces the inflammatory response through the downregulation of chemokines and cytokines secreted by keratinocytes 56. Prolonged inflammation causes the dysregulation of the sequential cascade of the wound healing process, leading to delayed wound healing 6. Prolonged inflammation is due to antagonism between anti-inflammatory factors and excess oxygen free radicals, causing delayed wound healing and subsequently leading to nonhealing chronic wounds 6. In the present investigation, EGF expression was elevated in the group with the DM wound treated with ADM-ASCs (DM + A/A), indicating epidermal regeneration and anti-inflammation in diabetic wound healing. VEGF is an important regulator of pathophysiological angiogenesis and vascular development in wound healing 57. VEGF expression was found elevated in the early phase of wound healing then followed with a decrease in the late phase 58-60. In the study of Li et al., VEGF expression was significantly weak during 4-8 weeks of wound healing stage 58. In the current study, on day 42 (end of the sixth week), no significant difference was observed in VEGF expression between all the experimental groups (Fig 6.). CD45 is a leukocyte marker expressed by fibrocytes, which are highly associated with macrophage-driven chronic inflammation 61, 62. In the current study, ADM-ASCs-treated rats (DM + A/A) showed decreased CD45 expression, indicating anti-inflammation in diabetic wound healing. Ki67 protein expression is highly associated with cell proliferation and regeneration in wound healing. In the current study, ADM-treated rats (DM + ADM) and ADM-ASCs-treated rats (DM + A/A) showed increased Ki67 expression, indicating elevated cell proliferation that ultimately enhanced the diabetic wound healing process. P4HA1 is an enzyme required for collagen biosynthesis 63. In the current study, ADM-ASCs-treated rats (DM + A/A) showed elevated P4HA1 expression, indicating collagen deposition in the diabetic wound. In summary, ADM, ABCcolla^®^ Collagen Matrix tissue-engineered, with ASCs (DM + A/A) enhanced wound healing by modulating the expression of EGF, VEGF, CD45, Ki67, and P4HA1 in diabetic rats and subsequently reorganizing the wound healing cascade that involves epidermal regeneration, anti-inflammation, collagen production and processing, and cell proliferation (Fig. 7).

### Limitations

In this study, there are three main limitations that need to be improved in future studies. First, the sample size should be larger for more experimental integrity. Second, head-to-head clinical comparison between human- and porcine-based ADM scaffolds decellularized through scCO_2_ is needed to determine which scaffold would exhibit more favorable performance for enhancing diabetic wound healing. Third, the results of the current study cannot be directly extrapolated to humans. The clinical benefits of autologous ASCs combined with the ADM for DM patients still need to be proven. In addition, it should be determined whether ASCs injected or seeded into ADM would result in more favorable clinical outcomes of wound healing.

## Conclusion

In the present study, scCO_2_-decellularized ADM scaffolds showed no obvious nucleus and DNA, confirming the complete decellularization of ADM scaffolds. ADM-ASCs enhanced cell proliferation and regeneration in diabetic wounds and thus accelerated the wound healing process. In addition, ADM-ASCs attenuated inflammation in diabetic wounds and thus promoted wound healing and tissue regeneration. Therefore, the ABCcolla^®^ Collagen Matrix dressing, which is ADM produced from porcine skin through scCO_2_ technology, seeded with ASCs had potential for accelerating diabetic wound healing in diabetic rats by modulating epidermal regeneration, anti-inflammation, collagen production, processing, and cell proliferation.

## Figures and Tables

**Table 1:** Scoring of the immunohistochemical expression of EGF, VEGF, CD45, Ki67, and P4HA1.

	DM- (a)	DM + ADM (b)	DM + ASCs (c)	DM + A/A (d)	*p* value b-a	*p* value c-a	*p* value d-a	*p* value b-c	*p* value b-d	*p* value c-d
EGF	49.62 ± 8.36	68.97 ± 7.49	59.03 ± 6.52	73.23 ± 8.03	0.048*	0.426	0.004**	0.372	0.283	0.092
VEGF	75.54 ± 4.39	61.73 ± 5.49	78.01 ± 5.73	77.66 ± 10.01	0.053	0.082	0.581	0.311	0.254	0.624
CD45	77.37 ± 8.40	60.08 ± 7.27	47.37 ± 7.56	45.27 ± 9.45	0.036*	0.008**	0.007**	0.524	0.362	0.852
Ki67	5.75 ± 3.48	25.06 ± 7.40	24.06 ± 6.68	38.02 ± 7.03	0.044*	0.041*	0.003**	0.084	0.061	0.059
P4HA1	40.30 ± 7.68	83.06 ± 10.38	66.45 ± 6.49	79.96 ± 8.23	0.005**	0.038*	0.004**	0.230	0.752	0.085

DM-: DM wound without treatments; DM + ADM: DM wound with acellular dermal matrix; DM + ASCs: DM wound with adipose stem cells; DM + A/A: DM wound with treatments with acellular dermal matrix and adipose stem cells; EGF: epidermal growth factor; VEGF: vascular endothelial growth factor; CD45: leukocyte marker associated with collagen production; Ki67: marker associated with cell proliferation and regeneration; P4HA1: prolyl 4-hydroxylase; *: *p* ≤ 0.05; **: p ≤ 0.01
